# Exercise training enhanced myocardial endothelial nitric oxide synthase (eNOS) function in diabetic Goto-Kakizaki (GK) rats

**DOI:** 10.1186/1475-2840-7-34

**Published:** 2008-11-19

**Authors:** James Grijalva, Steven Hicks, Xiangmin Zhao, Sushma Medikayala, Pawel M Kaminski, Michael S Wolin, John G Edwards

**Affiliations:** 1 Department of Physiology, New York Medical College, Valhalla NY, USA

## Abstract

**Background:**

Different mechanisms of diabetic-induced NO dysfunction have been proposed and central to most of them are significant changes in eNOS function as the rate-limiting step in NO bioavailability. eNOS exists in both monomeric and dimeric conformations, with the dimeric form catalyzing the synthesis of nitric oxide, while the monomeric form catalyzes the synthesis of superoxide (O_2_^-^). Diabetic-induced shifts to decrease the dimer:monomer ratio is thought to contribute to the degradation of nitric oxide (NO) bioavailability. Exercise has long been useful in the management of diabetes. Although exercise-induced increases expression of eNOS has been reported, it is unclear if exercise may alter the functional coupling of eNOS.

**Methods:**

To investigate this question, Goto-Kakizaki rats (a model of type II diabetes) were randomly assigned to a 9-week running program (train) or sedentary (sed) groups.

**Results:**

Exercise training significantly (p < .05) increased plantaris muscle cytochrome oxidase, significantly improved glycosylated hemoglobin (sed: 7.33 ± 0.56%; train: 6.1 ± 0.18%), ad improved insulin sensitivity. Exercise increased both total eNOS expression and the dimer:monomer ratio in the left ventricle LV (sed: 11.7 ± 3.2%; train: 41.4 ± 4.7%). Functional analysis of eNOS indicated that exercise induced significant increases in nitric oxide (+28%) production and concomitant decreases in eNOS-dependent superoxide (-12%) production. This effect was observed in the absence of tetrahydrobiopterin (BH_4_), but not in the presence of exogenous BH_4_. Exercise training also significantly decreased NADPH-dependent O_2_^- ^activity.

**Conclusion:**

Exercise-induced increased eNOS dimerization resulted in an increased coupling of the enzyme to facilitate production of NO at the expense of ROS generation. This shift that could serve to decrease diabetic-related oxidative stress, which should serve to lessen diabetic-related complications.

## Background

In the management of diabetes there is considerable evidence to demonstrate the benefits of exercise including improved glycemic control, an increased quality of life, and a reduction of cardiovascular risk factors. Exercise with and without dietary changes resulted in a significant reduction in glycosylated hemoglobin (HbA_1c_), increased insulin sensitivity, improved blood lipid levels, and lowered blood pressure [1,2]. Even low intensity forms of exercise such as walking will benefit NIDDM patients [1].

Exercise induces angiogenesis and altered vasculature reactivity in different vascular beds [3,4]. Exercise increases the sensitivity to endothelium-dependent relaxation by acetylcholine, but not the endothelium-independent response to sodium nitroprusside [3]. Chronic exercise increases NO production as early as one week after the start of training [4]. These changes are thought to be the result of increased eNOS protein [5,6]. Training effects may be limited to the vasculature of the working muscles; no effect was observed in mesenteric arterioles, suggesting that exercise-induced increases in stress may have be the responsible mechanism [7]. Several groups have reported that shear stress induces increases in eNOS expression [8,9]. However, studies in both diabetic patients and in diabetic animals have yielded different results; that vascular beds not participating in the response to exercise demonstrate significant improvements, suggesting that mechanisms other than localized stimuli are important [10,11].

Nitric oxide (NO) signaling regulates vascular tone, inhibits components of the atherogenic process, and influences myocardial energy consumption [12,13]. NO synthesis is governed by nitric oxide synthase (NOS). Three isoforms of NOS have been identified which are the products of three separate genes; endothelial NOS (eNOS), inducible NOS (iNOS), and neuronal NOS (nNOS). These isoforms share about 50–60% sequence identity and all use L-arginine, O_2_, and NADPH to catalyze the synthesis of NADP, citrulline, and NO as well as superoxide. Structural domain studies of the NOS molecule have identified separate oxygenase and reductase domains [14]. Dimerization is a requirement for catalytic activity of eNOS, although the truly active form is a complex that includes calmodulin, FAD, tetrahydrobiopterin (BH4), and iron protoporphyrin IX (haem) [14]. The dimeric form catalyzes the rate-limiting step in the synthesis of nitric oxide, while the monomeric form catalyzes the synthesis of O_2_^-^, a highly reactive oxidant species (ROS) [15]. The products catalyzed by eNOS are subject to complex regulation that we are just now beginning to understand. NO is an autocrine factor that regulates myocardial functioning via multiple mechanisms [16]. More recently Zhang et.al demonstrated that exercise training was associated with increased myocardial eNOS levels and enhanced myocardial contractility [17].

Different mechanisms of diabetic-induced NO dysfunction have been proposed and central to most of them are significant changes in eNOS function as the rate-limiting step in NO bioavailability. Several studies have reported decreased eNOS activity/protein levels in diabetic patients or animal models of diabetes [18-20]. The composition of the eNOS complex is critical for the relative formation of NO or superoxide formation. The mechanisms responsible for eNOS dysfunction remain unclear, however, a decrease in the dimer to monomer eNOS ratio within the myocardium of diabetic animals has been reported [15]. Although exercise-induced increases in eNOS expression have been documented, it is unclear if exercise may also alter the functional coupling of eNOS. To investigate this question, Goto-Kakizaki rats, a model of NIDDM, were exercise trained, to test if chronic exercise could improve eNOS function and enhance NO bioavailability.

## Methods

### Training Protocol

Twenty male GK rats were randomly assigned to exercise training (train) or sedentary (sed) groups. Rats were run on a motor driven treadmill set at a ten-degree incline. Animals were initially run at approximately 50% VO_2max _and the animals were run for up to 60 minutes and 5 days/week for 9 weeks. While training, animals were closely monitored to ensure animal safety and training compliance. Experimental protocols had institutional approval and animals were maintained in accordance with APS's *Guiding Principles in the Care and Use of Animals *and the *Guide for the Care and Use of Laboratory Animals*.

### Glucose Tolerance Test

Following an overnight fast, animals were injected with Nembutal (40 mg/kg *i.p*.). To perform the glucose tolerance test, sterile glucose (1.0 g glucose/kg *i.p*.) was injected into the abdominal cavity and tail vein blood sampled at selected intervals. Insulin was determined from plasma samples by ELISA (Crystal Chem, Downers Grove, IL). Following the glucose tolerance test, animals were given additional Nembutal (100 mg/kg *i.p*.) and sacrificed for tissue harvest.

### Low Temperature (LT) Electrophoresis and Western Blot Analysis

Tissues were stored at -80°C until used. LV samples were homogenized in ice-cold buffer (20 mM HEPES pH7.5, 50 mM NaCl, 1% SDS, 1× protease inhibitor (Sigma-Aldrich, P-8340). Protein concentration was determined by the Bradford method (BioRad reagent). LT electrophoresis is an in vitro test to determine the ratio of dimerized and monomerized forms of eNOS, with an increase in this ratio reflective increased coupling of the eNOS enzyme. For LT electrophoresis, samples were mixed with Lamelli buffer (without β-mercaptoethanol except where noted), before samples were loaded onto a 7.5% SDS-PAGE and electrophoresis was performed at 4°C. For total eNOS determinations, samples were mixed with Lamelli buffer, heated to 95°C for 5 min, and loaded onto a 7.5% SDS-PAGE and electrophoresis was perform at room temperature. The gels were blotted onto Hybond-P (Amersham Biosciences, Piscataway NJ) by a semi-dry transfer protocol. Western analysis was performed as described previously [21]. Antibodies used included eNOS (BD Transduction, San Jose, CA) and alpha-MHC derived from the BA-G5 cell line (ATCC, Manassas, VA). Antibody binding was visualized using the Amersham ECL *Plus *kit. Band density was quantified using AlphaEaseFC software (AlphaInnotech, San Leando CA). The dimer:monomer ratio calculated from band densities: % Dimerized eNOS = {dimer density/(dimer density + monomer density)}*100. Values presented are mean ± SEM.

### eNOS Activity

eNOS enzyme activity was determined by fluorescence spectroscopy method, using 2,3-diaminonaphthalene (DAN), and modified from that described by Misko et.al. [22]. In brief, LV was homogenized in ice-cold buffer (20 mM HEPES-pH 7.4, 0.1 mM EDTA, 1 mM glutathione, 10 μM BH_4_, 1× proteinase inhibitor. The homogenate was centrifuged at 1000 × g for 10 minutes at 4°C, and the supernatant retained for protein determination. To measure activity; 100 μg protein was incubated in reaction buffer (30 μM arginine, 1 μM FAD, 0.5 mM NADPH, 50 nM calmodulin). The reaction mixture was incubated for 3 hours at 37°C before the addition of acidified DAN. Fluorescence was determined using a Kontron SFM 25 spectrofluorometer (excitation 365 nm; emission 450 nm). eNOS specific activity was the difference in fluorescence between CaCl_2 _and calcium-free EGTA buffered solutions. To determine this, separate reactions were performed in the presence of 1 mM CaCl_2 _or 0.5 mM EGTA and the difference taken as eNOS specific activity. To examine the role of BH_4 _the reactions were also performed in the presence or absence of 7.5 μM BH_4_. Nitrite formation was calculated from standards using sodium nitrite. Using 750 nM 7-nitroindazole (a nNOS specific inhibitor) preliminary experiments indicated that nNOS did not contribute to left ventricular calcium-dependent NOS activity (data not shown). Values presented are mean ± SEM of arbitrary fluorescent units.

### Superoxide Activity

Superoxide activity was determined by two methods. In brief, LV was homogenized in ice-cold buffer (20 mM HEPES-pH 7.4, 0.1 mM EDTA, 1 mM glutathione, 10 μM BH_4_, 1× proteinase inhibitor. Protein concentration was determined by the Bradford method. NADPH oxidase activity, using 5 μM lucigenin, was determined as we have previously described [23]. NOX-dependent activity was determined by the addition of 300 μM apocynin. eNOS dependent superoxide activity was determined using dihydroethidium (DHE), as described by Zhao et.al. [24] To measure eNOS-driven superoxide activity, separate solutions containing either 10 μM BH_4 _or vehicle were added to reaction buffer (30 μM arginine, 1 μM FAD, 0.5 mM NADPH, 1 mM CaCl_2_, 50 nM calmodulin). 200 μg protein was incubated at 37°C and the development of fluorescence measured using an Infinite M200 plate reader (Tecan, Research Triangle Park, NC) (excitation; 480 nm, emission; 573 nm). Values presented are mean ± SEM of normalized arbitrary optical density (OD) units.

### Cytochrome Oxidase Assay

Cytochrome oxidase activity was determined as previously described [21]. The rate of cytochrome C oxidation was followed at 550 nm (ε = 21.0 mM^-1 ^cm^-1^), using an Ultrospec 3100 spectrophometer (Amersham Biosciences, Piscataway NJ).

### RNA Analysis

Total RNA from left ventricle was isolated using a FASTRNA *ProGreen *Kit (Q-Biogene, Irvine CA). Quantification of mRNA levels was done by QRT-PCR by real-time fluorescent using a Stratagene MX3000p as described previously [21]. The following genes were studied: αMHC (f;5'-CTACAAGCGCCAGGCTGAGG-3' ; r; 5'-GTGGGATAGCAACAGCGAGGC-3'), NADPH Oxidase 1 (f; 5'-TCCTCACTGGCTGGGATAGC-3' ; r; 5'-TTGAGTACCGCCGACAGCAT-3'), NADPH Oxidase 2 (f; 5'-TTGAGTGGTTCGCAGACCT-3' ; r; 5'-GTTGGGCCGTCCATACAG-3'), p47 (f; 5'-ATCCCAACTACGCAGGTGAA-3'; r;5'-TATCTCCTCCCCAGCCTTCT -3'), GTP cyclohydrolase (f; 5'-AAGGGTCCATATTGGTTATCTTCCT-3' ; r; 5'-ACACCTCGCATGACCATACA-3'), eNOS (f; 5'-GGCATACAGAACCCAGGATGG-3'; r; 5'-GCAGGCTGCAGTCCTTTGAT-3'), β-actin (f; 5'-GCGGTGACCATAGCCCTCTTT-3'; r; 5'-TGCCACTCCCAAAGTAAAGGGTCA-3'). The data was normalized by Δ2Ct method using β-actin, and the fidelity of the reactions was verified by melting point analysis. Data presented are the mean ± SEM with respect to sedentary control values. β-actin expression was compared to hypoxanthine phosphoribosyltransferase (HPRT) another "housekeeping" gene and no differences were observed.

### Statistical analysis

Statistical analyses were performed using NCSS Software (NCSS, Kaysville UT). Where appropriate, student t-test or ANOVA was utilized; post-hoc analysis was done using a Fisher's LSD analysis. Values presented are mean ± SEM, and statistical significance was set at p < .05.

## Results

Plantaris muscle cytochrome oxidase enzyme activity, a marker of aerobic metabolism and peripheral training adaptations was significantly increased following 9 weeks of training in the diabetic rats compared to sedentary controls (Table 1). Similarly, training significantly increased left ventricular weight (sedentary: 0.72 ± 0.02, train; 0.80 ± 0.01 g), while body weights were unaltered (Table 1). We have previously reported that high intensity exercise will increase left ventricular α-MHC [21]. In the present study using a lower intensity of exercise training, we did not observe a change in left ventricular α-MHC protein (sedentary; 100 ± 6.2%, Train; 100 ± 5.4%) or α-MHC-mRNA levels. Increases in skeletal muscle cytochrome oxidase activity and left ventricular weights are both indicative of training adaptations to endurance exercise.

**Table 1:** Morphometric and Blood Parameter Data

	Body mass	LV/BW	Plantaris Cytochrome Oxidase	Fasting Glucose	Fasting insulin	120 Insulin	ISI	HbA1c
	(g)		nmole/min/mg	(mg%)	(ng/ml)	(ng/ml)		(%)

Sedentary	384	1.86	0.31	205.3	2.57	4.56	16.6	7.36
	± 7	± 0.05	± 0.02	± 9.5	± 0.27	± 0.66	± 1.60	± 0.56

Exercise Trained	374	2.15	0.39	173.6	1.46	3.77	29.3	6.16
	± 5	± 0.04	± 0.05	± 9.5	± 0.17	± 0.59	± 4.5	± 0.16

	ns	p < .05	p < .05	p < .05	p < .05	ns	p < .05	p < .05

**Figure 5:**
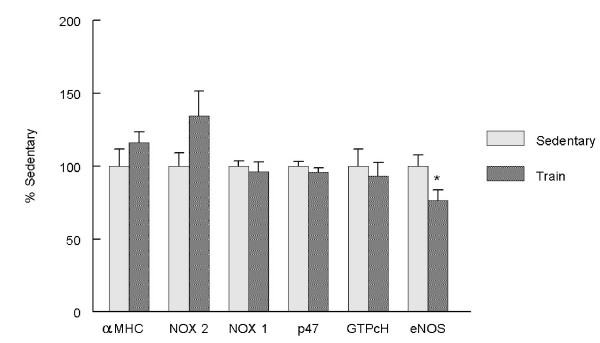
**Exercise training decreased LV eNOS-mRNA levels. Total LV RNA was isolated and quantification of mRNA levels was done by QRT-PCR as described in Methods.** Data was normalized by the Δ2Ct method using β-actin, and the fidelity of the reactions was verified by melting point analysis. Values presented are % control mean ± SEM of 10 animals per group; * p < .05 compared control.

**Figure 1:**
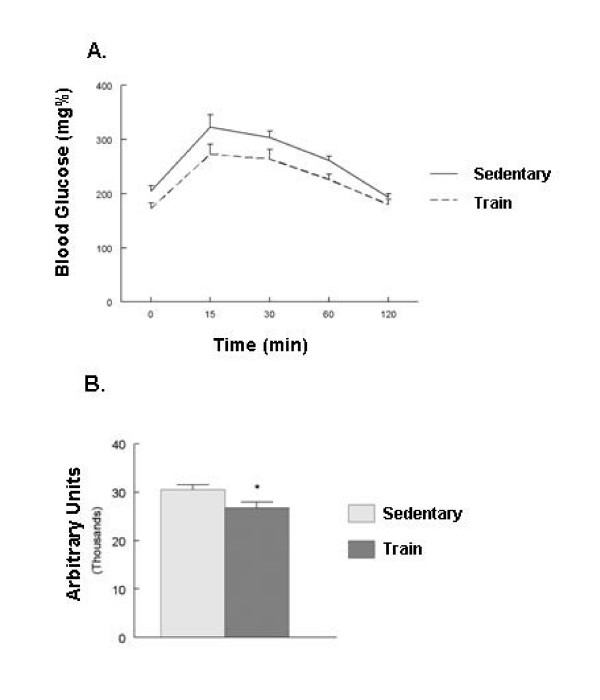
**Exercise improves glucose tolerance in the GK rats.** Sedentary and exercise trained GK rats were anesthetized and then injected with 1.0 g/kg glucose as described in Methods. A. Tail blood glucose wad determined at select intervals. B. Area under the curve analysis was performed using the NCSS software. Values are mean ± SEM of 10–14 animals; * p < .05 compared to control.

The exercise trained GK rats had a significant decrease in fasting blood glucose and consistent with this, a significant decrease in HbA1c (Table 1). Exercise has been useful in the management of diabetes in obese models of NIDDM, in part due to improved handling of blood glucose levels. To determine if training altered the sensitivity to glucose, a glucose tolerance test was performed. A significant decrease in the area under the curve analysis (AUC) in the trained animals was observed compared to sedentary animals (Figure 1). Plasma insulin levels were determined prior to the start of the glucose tolerance test and after 120 minutes. Fasting levels were significantly lower in the trained animals compared to sedentary animals (Table 1). Calculation of an insulin sensitivity index as described by Matsuda and DeFronzo, determined that exercise training significantly increased insulin sensitivity (Table 1) [25].

**Figure 2:**
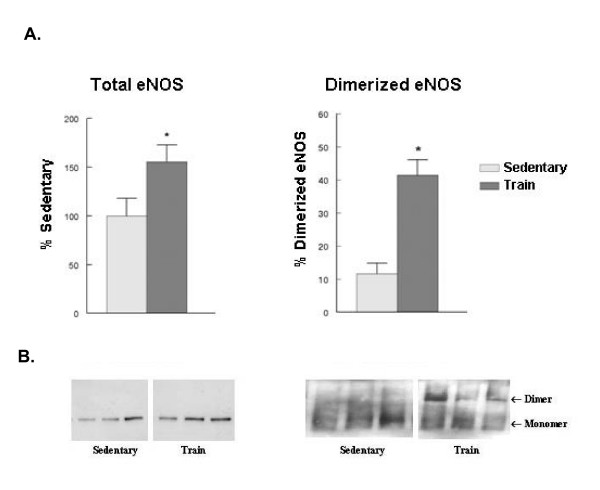
**Exercise training increased eNOS protein levels and eNOS dimerization.** Total protein from the left ventricle was prepared and electrophoresis under normal or low temperature conditions were carried out as described in Methods. A. Quantitative analysis of eNOS expression. Values are mean ± SEM of 6–10 animals per group; * p < .05 compared to control. B. Representative western blots of eNOS protein.

Exercise training has been reported to increase eNOS protein in different tissues [5,6]. Consistent with those findings, we observed a significant increase in left ventricular total eNOS (Figure 2). Dimerization of eNOS is required for the synthesis of NO and nitric oxide, and a decrease in the dimer:monomer eNOS ratio has been reported in diabetic myocardium [15]. When low temperature electrophoresis was performed under nonreducing conditions, we found that the ratio of dimer:monomer eNOS was also significantly increased by exercise training (Figure 2). When the samples were treated with 0.2% β-mercaptoethanol and low temperature electrophoresis was performed, the ratio of dimer:monomer eNOS was compressed but still significantly increased (sed: 17.8 ± 1.4%; train: 28.5 ± 4.1%). In most studies changes in eNOS have only been observed in those tissues undergoing exercise-induced increases in blood flow. In the present study total kidney eNOS levels tended to be increased, but a high level of variance blunted the statistical evaluation (sed; 100 ± 11.1%; train: 151.5 ± 22.3%: p = 0.06). However, a significant increase in the dimer:monomer ratio was observed in the eNOS protein from kidney (sed: 12.0 ± 1.7%; train: 20.7 ± 2.9%). Thus exercise training increased dimerization of eNOS in both tissues.

**Figure 3:**
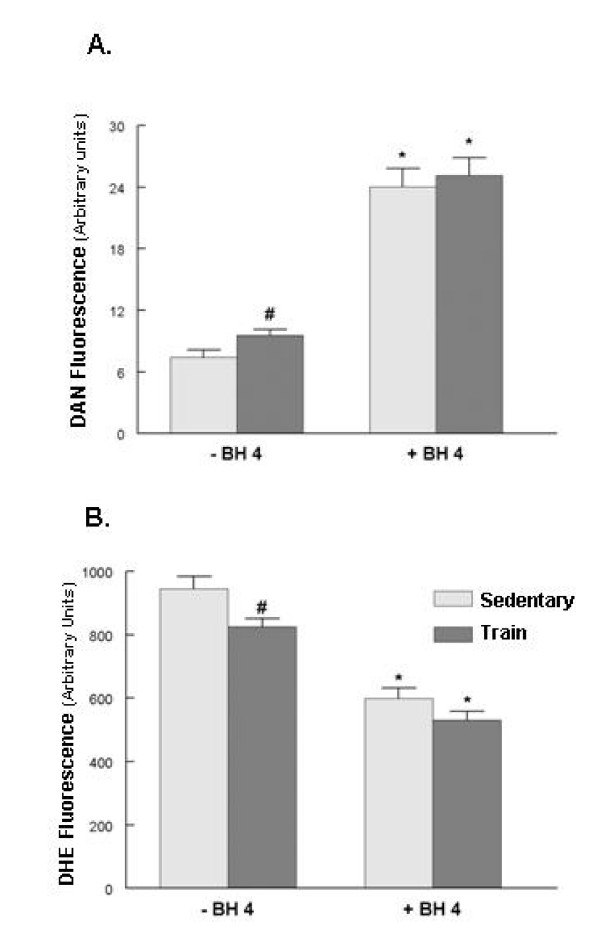
**Exercise training enhances eNOS function in the absence of BH_4_.** A. eNOS activity was measured using in the absence or present of BH_4_. B. eNOS-dependent superoxide formation was measured as described in Methods. Values are mean ± SEM of 10 animals per group; # p < .05 compared to respective control, * p < .05 compared to respective -BH_4 _assay group.

**Figure 4:**
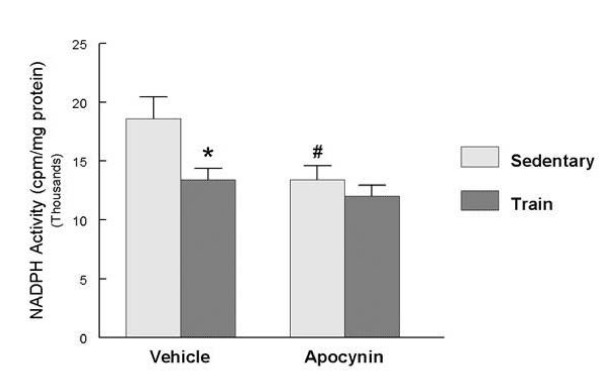
**Exercise training decreased LV superoxide generation.** LV homogenates were prepared as described in Methods to determine superoxide generation. Activity was assay in a Krebs-HEPES buffer using 5 μM lucigenin, 200 μM NADPH and in the absence or presence of 300 μM apocynin. Values are mean ± SEM cpm/mg protein of 10 animals per group; # p < .05 compared to respective control, * p < .05 compared to respective -BH_4 _assay group.

Increased dimerization of the eNOS protein should shift its enzymatic activity towards improved NO generation, at the expense of superoxide production. To examine this concept, two in vitro determinations of eNOS function were made. In the absence of exogenous BH_4_, exercise training significantly increased eNOS activity compared to sedentary controls (Figure 3A). The addition of exogenous BH_4 _to the reaction buffer significantly increased eNOS activity in both groups, but canceled the differences between the groups. Determination of eNOS-driven superoxide generation found that in the absence of added BH_4_, exercise training resulted in significantly decreased superoxide production compared to the sedentary group (Figure 3B). The addition of BH_4 _to the reaction media decreased eNOS-driven superoxide generation in both groups and no difference between sedentary and exercise trained groups were observed. Exercise training also significantly decreased ventricular NADPH oxidase activity (Figure 4). In the presence of the NADPH oxidase inhibitor apocynin, NADPH oxidase activity was significantly decreased only in the sedentary group and the differences between sedentary and trained groups were eliminated.

The apparent shift in NADPH oxidase activity suggests that this enzyme complex is either less active or that exercise decreased expression of the constitutive subunits. However, when expression of three subunits (NOX1, NOX2, and p47) was examined no significant changes in the relative mRNA levels were observed between the sedentary and exercised trained groups. Similarly, expression of GTP cyclohydrolase, the rate limiting step in BH4 synthesis, was unaltered by exercise training. In contrast to increased levels of eNOS protein, exercise training did cause a significant decrease in eNOS-mRNA levels (Figure 5).

## Discussion

Even low intensity forms of exercise such as walking have been shown to benefit NIDDM levels, and patients [1]. Exercise lowers HbA_1c_, increases insulin sensitivity, improves blood lipid levels, and lowers blood pressure in diabetic individuals [1,2]. Exercise-induced increases in tissue eNOS expression have been reported and our results in the heart are consistent with those findings. The major finding of this study was that exercise training increased eNOS function, in part by an increase in dimerization, as one mechanism for increasing NO bioavailability in the diabetic heart.

GK rats have elevated fasting glucose (150–250 mg%), are hyperinsulinemic at a young age, have impaired response to glucose, and increased HbA1c [26]. As a model of type 2 diabetes, the GK rats do not have the confounding factors of hyperlipidemia or hypercholesterolemia observed in obese-diabetic rats. The etiology of the GK rats is unknown, but it has been suggested that impaired pancreatic mitochondrial function may partially explain the depressed insulin release [27]. GK rats display symptoms associated with diabetic complications, including reduced nerve conduction velocity indicative of peripheral neuropathy [28]. Progressive renal involvement in GK rats presents in a manner similar to NIDDM in humans; that includes thickening of the glomerular basement membrane [29]. Similar to others, we have also found endothelium-dependent microvascular dysfunction in the GK rats (data not shown) [26,30,31]. GK animals also display increases in oxidative stress markers and susceptibility to lipid peroxidation in the hearts of older (12–18 months) but not younger (3–6 months) animals [32].

Exercise-induced increases in vascular eNOS protein have been known for sometime [5,6,33]. More recently, it has been shown that exercise will also alter myocardial eNOS protein and phosphorylation status [17]. Zhang et.al exercise trained rats by a 10 week swimming program [17]. They demonstrated that exercise increased myocardial NOx production, eNOS protein levels, and increased the sensitivity to insulin-stimulated phosphorylation of eNOS. The shift in eNOS phosphorylation (ser1179) status was mediated through the Akt signaling pathway and resulted in enhanced myocardial contractility [17]. Shifts in phosphorylation status do not fully explain our findings of increased eNOS dimerization. eNOS has 5 phosphorylation sites; serines 116, 617, 635, 1179 and threonine 497 (bovine coordinates). Whereas phosphorylation of the serines 617, 635 and 1179 will activate eNOS, the Thr^497 ^phosphorylation appears to serve as an intrinsic switch to enhance coupling of eNOS in favor of NO production at the expense of superoxide [34]. To date no one has reported exercise-induced alterations in eNOS-Thr^497 ^phosphorylation status. Fulton et al demonstrated that phosphorylation of ser1179 was not required for correct intracellular localization [35]. eNOS differs from iNOS and nNOS in that the former contains the consensus sequences for both myristoylation and palmitoylation. Myristoylation is required for initial targeting of eNOS to the cell membrane, while palmitoylation may stabilize the eNOS membrane association [36,37]. To date no one has reported exercise-induced changes in myristoylation or palmitoylation. However, the extent of site specific phosphorylation is modified by subcellular localization and hence stimulus specific activation that is dependent upon localization would be a useful avenue for future work [38].

Exercise improved the eNOS coupling state; a shift that could serve to decrease diabetic-related oxidative stress and lessen diabetic-related complications. Two *in vitro *determinations of eNOS function were made. Both measures were influenced by the addition of exogenous BH_4 _to the reaction. eNOS coupling was greater in the left ventricle from the exercise trained group only in the absence of exogenous BH_4_. This suggests that BH_4 _may have been more tightly bound to eNOS in the trained group or eNOS had an increased sensitivity to residual BH_4_, producing a shift in eNOS functional state.

BH_4 _is both an anti-oxidant and an essential cofactor of eNOS and other members of the monooxygenase family [39]. In both healthy individuals and diabetics, a single oral glucose challenge can reduce NO-specific vasodilatation, an action that was blocked by supplementation with tetrahydrobiopterin (BH_4_) [40-42]. Although the exact mechanism of its actions are not clear, it is thought that BH_4 _stabilizes the dimer conformation and facilitates L-arginine binding as the first step in its conversion to citrulline [43,44]. Shifts in BH_4 _concentration directly influence the rate of NO or superoxide anion O_2_^- ^production [39,45,46]. Both IDDM and NIDDM animal models have reported decreased concentrations of BH_4 _in the vasculature [47,48]. Differential effects of BH_4 _have also been observed with exercise in aged humans. Eskura et.al demonstrated that BH_4 _supplementation improved vasodilatation in sedentary but not exercise trained elderly humans, suggesting that chronic exercise improved BH_4 _bio-availability in that population [49]. We have demonstrated that exercise training improved eNOS function when BH_4 _may have been limiting. This suggests that chronic exercise enhanced eNOS function, in part through altered BH_4 _bioavailability.

We observed that exercise training reduced apocynin-sensitive chemiluminescence, indicative of a decrease in NAD(P)H oxidase activity in the left ventricle. Apocynin is thought to interfere with p47 activation of NAD(P)H oxidase, but more recently has also been suggested to act as an antioxidant [50,51]. If this latter mechanism was operational, we should have observed some decreases in both sedentary and training groups. However no differences were observed in the exercise trained group suggesting that chronic exercise modified the NAD(P)H oxidase complex activity. Plasma levels of angiotensin II (Ang II) are elevated in diabetics with poor glucose control and in untreated diabetic animal models [52-55]. Conversely, normalization of Ang II levels can be achieved with improved glucose handling or ACE inhibition [52,56]. Chronically elevated Ang II levels are associated with increased expression of NAD(P)H oxidase components and oxidative stress [56]. We did not observe changes in NOX 1, NOX 2, or p47-mRNA levels suggesting that altered expression was not influenced by training and that a shift in the activation of NADPH oxidase may have had a greater role in the observed improvements. ROS promotes oxidation of BH_4 _to dihydropterin and one consequence of decreased NAD(P)H oxidase activity may be to improve BH_4 _bioavailability [57].

Control of eNOS protein levels is a complex operation that is mediated on several levels including eNOS transcription, mRNA stability, and post-translational modifications. We have made the paradoxical observation that exercise training of diabetic animals lead to an increase in eNOS protein, but also a decrease in eNOS-mRNA. This outcome is the reverse observed by others who found that in comparison to controls, diabetes decreased eNOS protein but increased eNOS-mRNA [18,58]. Similarly, in cultured endothelial cells, elevated glucose levels increased eNOS-mRNA [59]. This paradox may be explained in part by diabetic-induced decreases in eNOS function which activates compensatory mechanisms. Drummond et.al. demonstrated that exogenous H_2_O_2 _increased eNOS transcription and eNOS-mRNA stability [60]. Our observation that exercise training decreased both eNOS-driven and NADPH oxidase derived ROS should serve to lessen this driving force for eNOS transcription. Second, hyperglycemia acts as a NO scavenger and the exercise-induced improvements in glycemic control should improve NO bioavailability [61]. Third, increased NO bioavailability is known to serve as a negative feedback mechanism to decrease eNOS transcription via a cGMP-mediated pathway [62,63]. Thus the decrease in eNOS-mRNA may have been the result of the decreased driving forces for eNOS transcription.

## Summary

**Figure 6:**
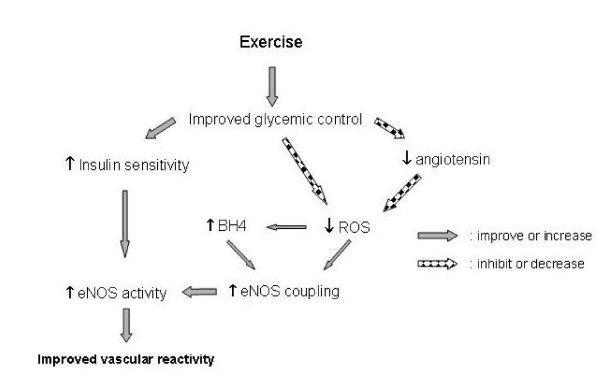
**Exercise induced alterations in the diabetic myocardium leading to improvements in eNOS function. **; decrease in level or inhibition of function, ; increase in level or improvement of function. Improvements derived from increased insulin sensitivity are from the findings of Zhang et.al. [17].

Regulation of eNOS function is showing itself to be an increasingly complex event that diabetes clearly disrupts. Exercise-induced increases in eNOS expression have been demonstrated in several reports as well as exercise-induced shifts of eNOS phosphorylation status. The major findings of this study are that exercise at least partially reversed diabetic-induced eNOS dysfunction through increased dimerization leading to increased NO generation at the expense of superoxide production. As shown in Figure 6, it is likely that this was achieved through several distinct mechanisms that converge on the management of cellular ROS and BH_4 _metabolism.

## Competing interests

The authors declare that they have no competing interests.

## Authors' contributions

JG and SH trained animals, performed data collection, assisted in data analysis and help write the manuscript, X.Z. and S.M. performed data collection and assisted in data analysis, P.K. and M.W. assisted in assay development, J.E. designed and coordinated the study and wrote the manuscript. All authors have read and approved the manuscript.
